# A robust CRISPR interference gene repression system in *Vibrio parahaemolyticus*

**DOI:** 10.1007/s00203-023-03770-y

**Published:** 2023-12-26

**Authors:** Taoyuan Jiang, Yuhuan Li, Wencong Hong, Mingyu Lin

**Affiliations:** 1 Department of Respiratory Medicine, Nan’an Hospital, 330, Ximei Residential District, Xinhua Street, Quanzhou, Fujian Province China; 2 The Second People’s Hospital of Three Gorges University, 18, Tiyuchang Road, Yichang, Hubei Province China

**Keywords:** CRISPRi, Essential gene, Gene repression, *Vibrio parahaemolyticus*

## Abstract

*Vibrio parahaemolyticus* is a significant cause of seafood-associated gastroenteritis and pestilence in aquaculture worldwide. Despite extensive research, strategies for protein depletion in this pathogen remain limited. Herein, we constructed a new CRISPR interference (CRISPRi) system for gene repression based on the combination of a shuttle vector pVv3 and the nuclease-null Cas9 variant (dead Cas9, or dCas9) from *Streptococcus pyrogens*. This CRISPRi is induced by adding both IPTG and arabinose. We showed that gene repression is scalable via the use of multiple sgRNAs. We also demonstrated that this gene repression can be precisely tuned by adjusting the amount of two different inducers and can be reversed by removing the inducers. This system provides a simple approach for selective gene repression on a genome-wide scale in *V. parahaemolyticus*. Application of this system will dramatically accelerate investigations of this bacterium, including studies of physiology, pathogenesis, and drug target discovery.

**Supplementary Information:**

The online version contains supplementary material available at 10.1007/s00203-023-03770-y.

## Introduction

*Vibrio parahaemolyticus* is a halophilic, gram-negative *γ*-proteobacterium that thrives in estuaries and marine and coastal environments (McCarter 1999). This bacterium is often planktonic but is also found on the surfaces of aquatic organisms (Gode-Potratz et al. 2011). Although most *V. parahaemolyticus* strains are not pathogenic, some strains acquire virulence factors, become causative agents (Boyd et al. 2008; Hubbard et al. 2016), and impose a massive economic burden on the shrimp industry (Tran et al. 2013). In addition, this bacterium is the leading cause of food-borne infections associated with acute gastroenteritis in the United States (Kaysner et al. 1990; Newton et al. 2012) and Asia (Alam et al. 2002).

Extensive research has been performed to decipher the molecular mechanism underlying *V. parahaemolyticus* infection, of which gene knockout plays a crucial role (Burdette et al. 2008; Hubbard et al. 2016; Li et al. 2019). However, bacteria cannot survive without essential genes; hence, gene knockout cannot be used in research on the functions of essential genes. Protein depletion is an alternative. It is widely used in the study of functions of essential genes, investigations of pathogenesis, and drug discovery (Gopal & Dick 2020; Powell et al. 2021; Wu et al. 2020). There are a few techniques that can be used for protein depletion in bacteria. The first is replacing the original promoter of the gene of interest (GOI) with an inducible promoter. The cells should be grown with induction to study the essential genes, and then protein depletion can be achieved by removing the inducers(de Lorenzo et al. 1993). The second is to tag the gene of interest (GOI) with the ClpXP recognition peptide; then, the protein is degraded by ClpXP machinery(Castang et al. 2008). The third is to introduce antisense RNA that targets the GOI (Magistro et al. 2018). However, these techniques cannot be used to control the timing and dynamics of protein depletion or tune the protein expression level, which prevents the study of essential genes. Furthermore, promoter replacement and ClpXP recognition peptide tagging are recombination-based techniques. Construction of the recombinant bacterial strains is time-consuming and laborious; CRISPR/dCas9-based gene interference (CRISPRi) provides an alternative (Zhang et al. 2021). This new technique enables protein depletion in bacteria via a convenient, versatile strategy.

The type II CRISPR/Cas9 system from *Streptococcus pyogenes* was re-engineered as a powerful tool for gene knockout and genome engineering (Cho et al. 2013; Shalem et al. 2014). In this system, a single-strand RNA (sgRNA) containing an ~ 20-nucleotide programmable RNA segment (called protospacer), which is complementary to an ~ 20-nucleotide target DNA segment of the genome, complexes with the Cas9 nuclease protein and directs this complex to the specific locus of genomic DNA by base pairing between the protospacer and the target DNA segment. The Cas9 nuclease cleaves the genomic DNA at the locus, generating a double-strand DNA break. The DNA break is repaired through nonhomologous end joining (NHEJ), but an indel is introduced, and the gene is then knocked out and inactivated (Jiang and Doudna 2017).

The CRISPR/Cas9 system from *S. pyogenes* is widely used in gene regulation (Adli 2018). First, the Cas9 protein is deactivated by introducing two point mutations in the RuvC1 and HNH domains (i.e., D10A and H840A). Then, a designed sgRNA binds deactivated Cas9 (dCas9) and directs the dCas9:sgRNA complex to the targeting sequence on genomic DNA. The dCas9:sgRNA complex blocks transcription initiation when directed to the promoter region by sgRNA. It disrupts transcription elongation when directed to the noncoding strand of the gene of interest, implementing gene repression (Choudhary et al. 2015; Cui et al. 2018; Depardieu and Bikard 2020; Gilbert et al. 2013; Jiang et al. 2013; Peters et al. 2016; Wong and Rock 2021). This CRISPRi method is programmable and highly efficient. CRISPRi is a powerful tool for studying the functionality of genes and the mechanism of pathogenesis; it has great potential in discovering functionally essential genes in bacteria (Zhang et al. 2021). Thus, the application of CRISPRi will further studies of bacterial physiology and pathogenesis, and discovery of new drugs (Fellmann et al. 2017; Rock 2019). However, no CRISPRi systems are available for *V. parahaemolyticus*, although this bacterium poses serious health hazards and industrial and agricultural losses. Thus, a CRISPRi system is urgently needed.

Cryptic plasmids can be isolated from *V*. *parahaemolyticus* (Guerry and Colwell 1977), and they can be engineered into a shuttle vector for convenient cloning (Datta et al. 1984; So et al. 2014). pVv3 is a shuttle vector derived from *Vibrio vulnificus*. It can be stably maintained in *V. parahaemolyticus* and efficiently expresses EGFP (Klevanskaa et al. 2014), demonstrating its ability to express exogenous proteins. Herein, we re-engineered pVv3 and constructed the plasmid pJT3 that expressed a (dCas9 and sgRNA-based) CRISPR interference (CRISPRi) system in *V. parahaemolyticus*. We showed that this system repressed gene expression efficiently when both IPTG and arabinose were available. This gene repression could be reversed via the removal of these two inducers and can be precisely and timely tuned by adjusting the amount of the inducers. We also demonstrated that gene perturbations by this CRISPRi are scalable when multi-sgRNAs were used, thereby showing great potential for research on *V. parahaemolyticus*.

## Result

### Construction of the CRISPRi system and transformation into *V. parahaemolyticus*

**Fig. 1:**
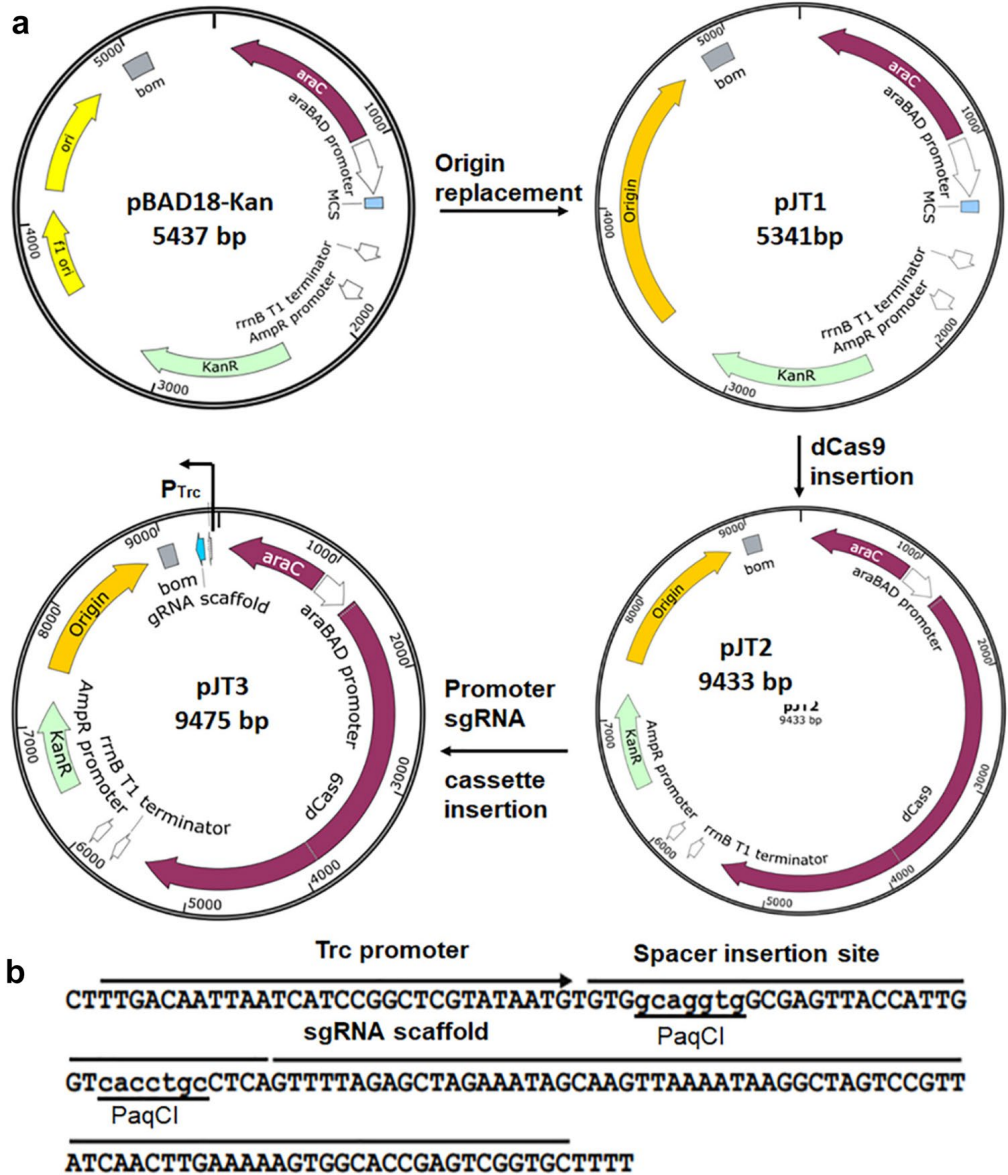
Maps and cloning sites of the CRISPRi system. **a** Schematic diagram outlining the construction of the CRISPRi plasmid used in this study. The replication origin of plasmid pBAD18-Kan was substituted with that of pVv3, and then the dCas9-coding gene and sgRNA transcription cassette were installed in opposite orientations. **b** Sequence of the protospacer cloning sites of the CRISPRi plasmid pJT3. A sgRNA expression cassette has two PaqCI sites for protospacer cloning, and sgRNA is driven by the Trc promoter

The CRISPR interference system (CRISPRi) was first shown to repress gene transcription in *E. coli* (Qi et al. 2013), which shows its strong potential in genomic functional studies in bacteria. Furthermore, it has been shown to function well as a transcriptional repressor or activator in many bacteria (Ganguly et al. 2020; Ho et al. 2020; Lunge et al. 2020). To develop an efficient and convenient CRISPR interference system for gene interference in *V. parahaemolyticus*, a single plasmid expressing dCas9 protein and sgRNA was constructed (Fig. 1), in which the origin of replication of pBAD18-Kan was replaced with the origin of replication of pVv3. The *S. pyogenes* CRISPR/dCas9-coding sequence was placed under the control of the P_Bad_ promoter (Guzman et al. 1995), and then the sgRNA expression cassette was placed under the control of the Trc promoter (de Boer et al. 1983). The sgRNA expression cassette has two PaqCI cutting sites, which facilitate the cloning of sgRNA sequences between them. To study the efficacy of repression of the combination of 2 sgRNAs, a J23119-driven sgRNA expression cassette was cloned and inserted into pJT3 to produce the plasmid pJT4 (SI Fig. 1).

Electroporation efficiently introduces plasmid DNA into *V. parahaemolyticus* (Hamashima et al. 1990). Restriction-modification (RM) systems protect bacteria from exogenous DNA. Methylation of plasmid DNA improves the transformation efficiency of exogenous plasmids. Before transformation, pJT3 plasmids were methylated as described by Donahue et al. (Donahue et al. 2000) with minor modifications before electroporating into *V. parahaemolyticus* cells. The results showed that methylation protected pJT3 from SalI digestion (SI Fig. 2) and improved transformation efficacy. The transformation efficiency increased by ca. fivefold (data not shown). Therefore, CRISPRi plasmids were routinely methylated before being transformed into *V. parahaemolyticus*.

**Fig. 2:**
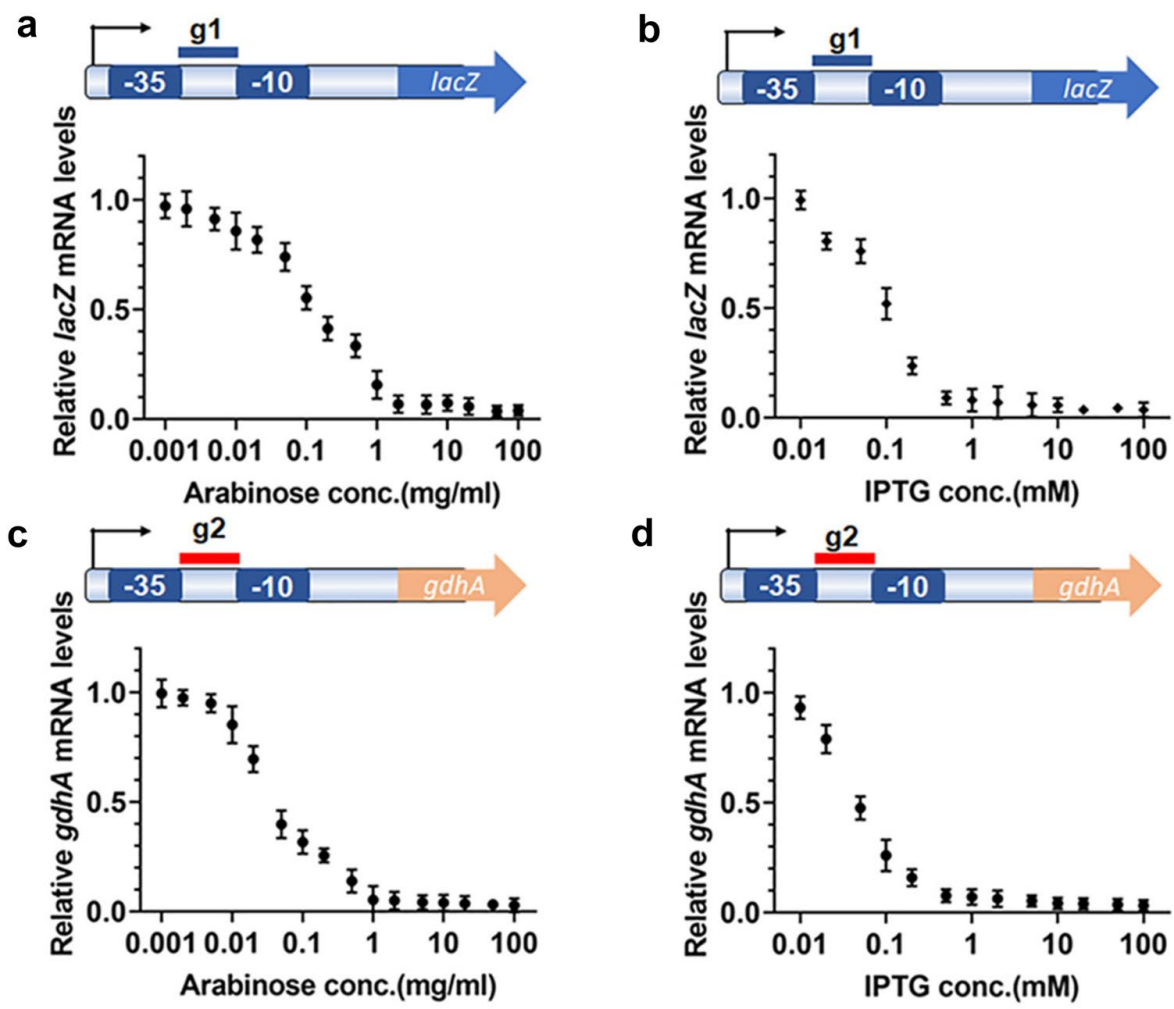
CRISPRi exerts robust gene suppression in *V. parahaemolyticus*. *lacZ* and *gdhA* mRNA were measured as a function of inducer (arabinose and IPTG) concentrations. mRNA levels were reported as relative values normalized to the levels of control samples. 2 mg/ml arabinose nearly obtained the maximal silencing effect and decreased mRNA by ca. 35-fold (**a** and **c**); 0.5 mM IPTG almost induced the maximal repression and reduced mRNA levels by ca. 25-fold (**b** and **d**). Experiments were performed on at least three biological repeats

### Optimizing inducer concentrations for gene silencing

To optimize inducers’ concentrations in gene repression by the pJT3 CRISPRi system in *V. parahaemolyticus*, we designed sgRNA g1 and g2, targeting the promoter regions of endogenous β-D-galactosidase encoding gene *lacZ* and glutamate dehydrogenase encoding gene *gdhA* in the bacterial chromosome, respectively (Fig. 2 and SI Table 1). Then, we cloned them into pJT3 and evaluated their repression efficiencies by titrating IPTG and arabinose successively. First, we induced sgRNA transcription by 1 mM IPTG and induced dCas9 protein expression with titrated arabinose concentrations for 6 h, and then we measured mRNA levels. We compared the mRNA levels of cells cultured in media with inducers to those cultured in media without any inducer.

We found that mRNA levels were titratable. More than 97% of the transcription of *lacZ* and *gdhA* was repressed at 100 mg/ml arabinose in LB medium with 1 mM IPTG (Fig. 2a, c). However, the transcription of most mRNA (96%) decreased at 2 mg/ml arabinose in the LB medium. Next, we induced dCas9 protein expression with 2 mg/ml arabinose, while inducing the transcription of sgRNA with titrated IPTG concentrations for 6 h. The mRNA levels of *lacZ* and *gdhA* decreased by 97% with 100 mM IPTG and were reduced to almost the lowest level (4%) at a lower concentration (0.5 mM) of IPTG (Fig. 2b, d). β-D-galactosidase activity assays were performed to confirm the repression; the results showed that 0.5 mM IPTG and 2 mg/ml arabinose reduced the enzymatic activities to almost the lowest levels (SI Fig. 3), which is consistent with the RT-qPCR results. Taken together, the results indicated that this CRISPRi system is sensitive and titratable upon the availability of inducers, i.e., arabinose and IPTG, and maximal gene repression can be achieved with 2 mg/ml arabinose and 0.5 mM IPTG.

**Fig. 3:**
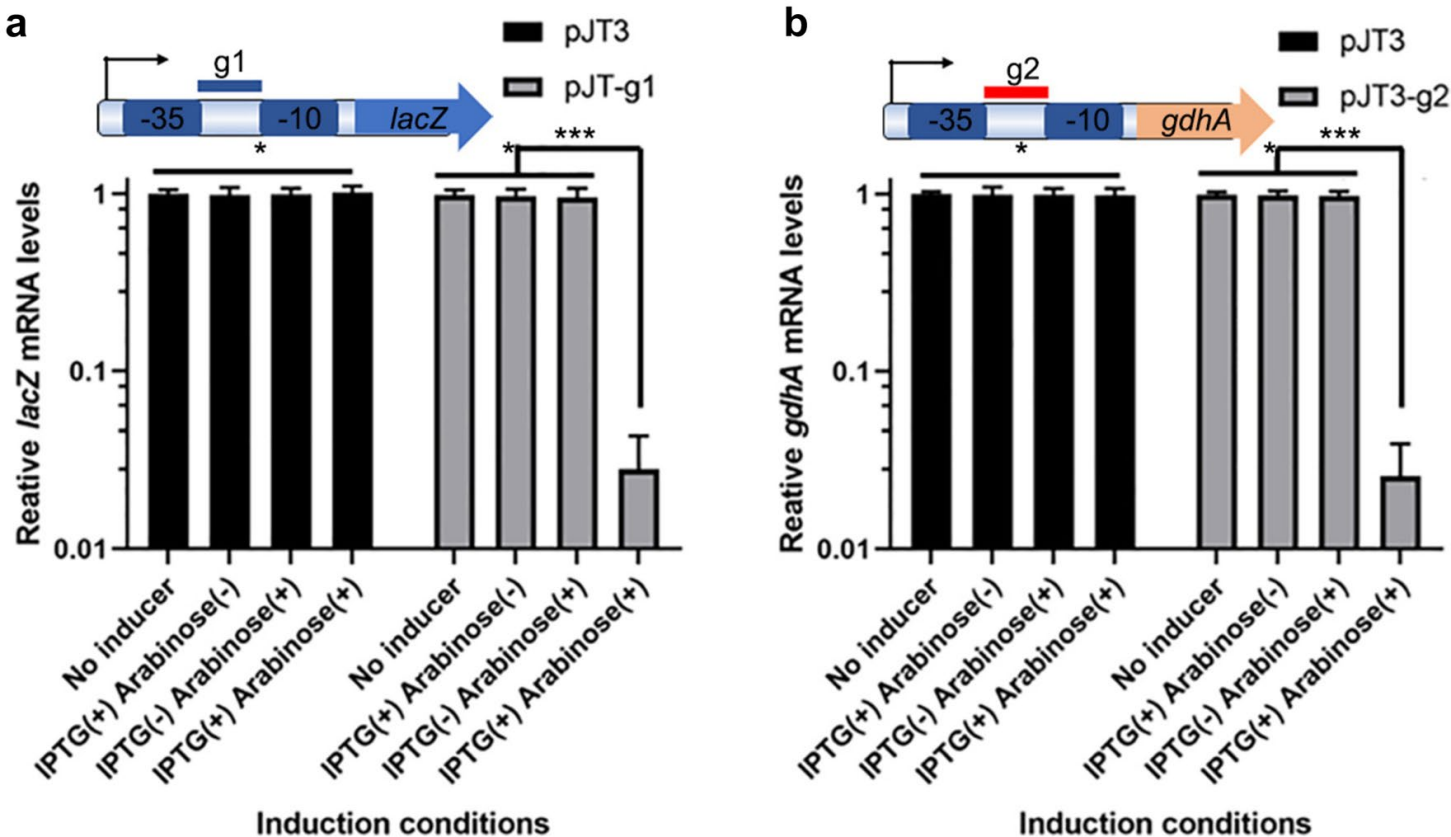
CRISPRi represses gene transcription in *V. parahaemolyticus*. Adding only one inducer repressed less than 5% of *lacZ* of *gdhA* transcription, while adding both inducers suppressed more than 95% of gene transcription. **a** Test of the leaky transcription of *lacZ* with arabinose and IPTG added. **b** Test of the leaky transcription of *gdhA* with arabinose and IPTG added. *P > 0.05, ***P < 0.05, Experiments were performed with at least three biological repeats. A *t* test was used to examine significance

When examining the possible leaked expression of sgRNA and Crispr-dCas9, we measured the mRNA levels of *V. parahaemolyticus* containing the pJT3 plasmid (as the control), pJT3-g1 plasmid (to examine *lacZ* mRNA levels) or pJT3-g2 plasmid (to examine *gdhA* mRNA levels). When a single inducer was presented, either 2 mg/ml arabinose or 0.5 mM IPTG, the transcription of *lacZ* and *gdhA* was almost completely ‘turned on’, and less than 5% of the transcription was repressed, showing virtually no expression of sgRNA or dCas9 when the corresponding inducer was not added. In contrast, when both 0.5 mM IPTG and 2 mg/ml arabinose were added to the cultures, the transcription of *lacZ* and *gdhA* was almost totally ‘turned off’, and less than 5% of *lacZ* and *gdhA* mRNAs were expressed, indicating that sgRNA and dCas9 were expressed efficiently (Fig. 3). This result showed that inducers tightly controlled the expression of the CRISPRi system (dCAS9 and sgRNA), and the CRISPRi system tightly controlled the transcription of the gene of interest.

### Optimizing sgRNA sequences with nonessential genes in *V. parahaemolyticus*

**Fig. 4:**
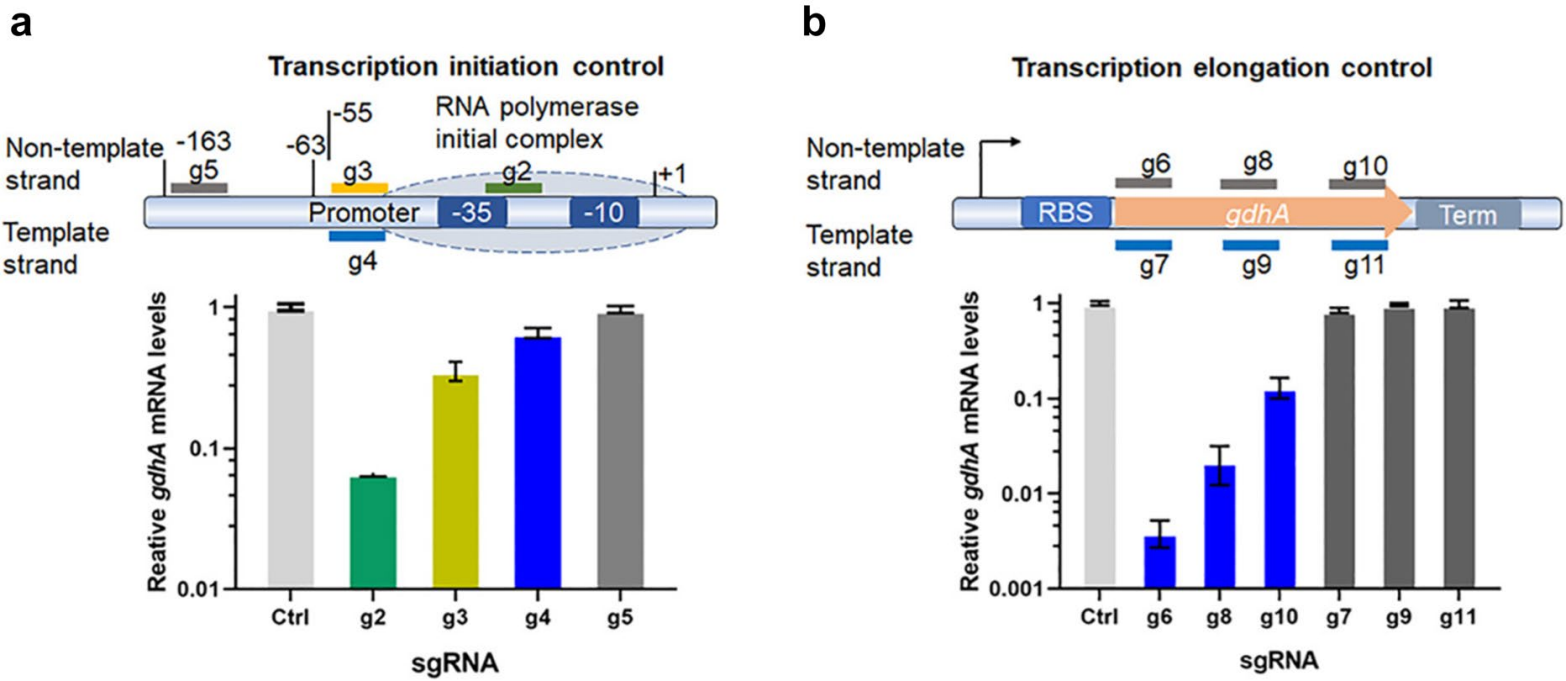
CRISPRi repressed gene transcription is locus and strand specific. **a** Test the repression efficiency of sgRNA targeting the promoter region. sgRNA targeting the sequence between the − 35 box and − 10 box suppressed gene transcription efficiently but less efficiently when targeting the sequence on the template strand. There was no effect when targeting the sequence 163 bp upstream of the TSS. **b** Test the repression efficiency of sgRNA targeting the transcription elongation region. sgRNA targeting the sequence close to the translation initiation site suppressed gene transcription more efficiently, while having almost no effect when targeting the sequence on the template strand

An optimal sgRNA sequence is critical for efficient CRISPRi repression (Qi et al. 2013). To optimize the sgRNA sequence for effective silencing using the pJT3 system, we designed one set of sgRNAs targeting different locations on the *gdhA* gene in *V. parahaemolyticus*: the promoter region, template strand, and non-template strand. We cloned them into pJT3 and examined their silencing efficiencies in *V. parahaemolyticus*. The results showed that sgRNA locations were crucial for effective silencing. Regarding the promoter sequence, a sgRNA (g2) targeting the sequence between the -35 box and the -10 box efficiently knocked down gene expression. In contrast, sgRNAs (g3 and g4) targeting the adjacent regions of the promoter sequence slightly reduced transcription. sgRNA (g5) targeting the sequence 163 bp upstream of the TSS did not have a noticeable effect on gene repression. When examining the repression efficiencies of sgRNAs targeting protein-coding regions, we found that sgRNAs (g6, g8, g10) targeting sequences on the non-template strand repressed transcription much more efficiently than those (g7, g9, g11) targeting sequences on the template strand, and the sgRNA targeting locus (g6) close to the translation initiation codon ATG was more efficient than the sgRNA (g10) targeting locus far away from ATG (Fig. 4). In other words, the distance between the ATG site and the sgRNA targeting sequence on the non-template strand is inversely correlated with repression efficiency.

**Fig. 5:**
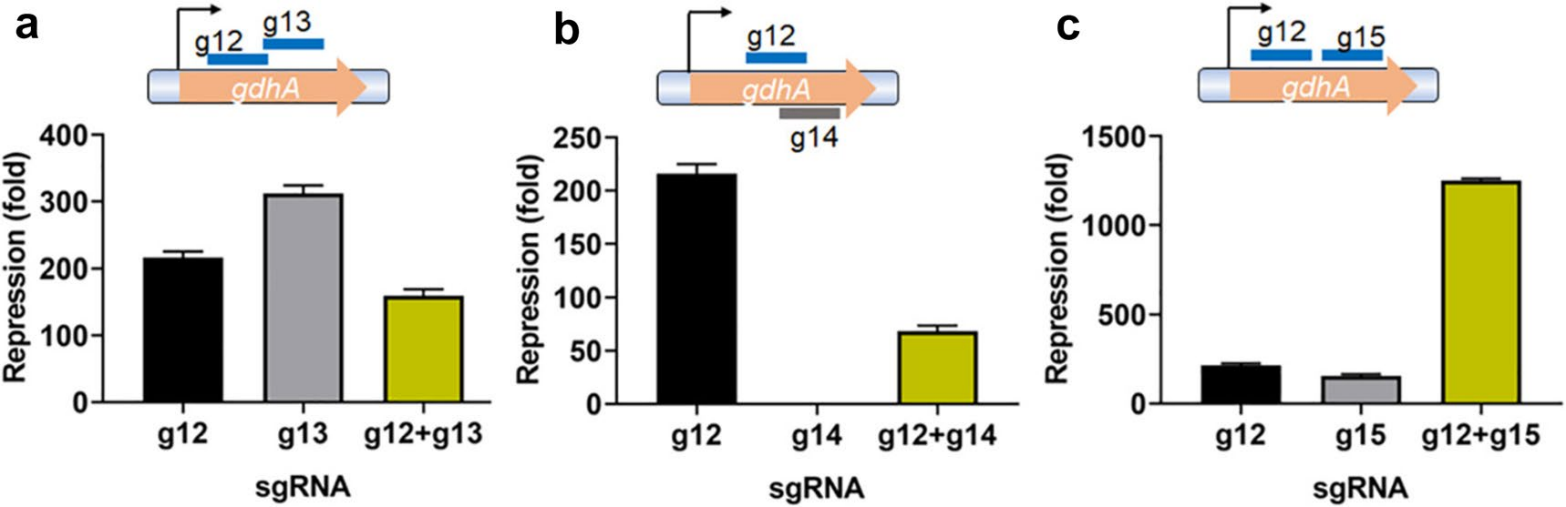
Testing the combinatorial silencing effects of using two sgRNAs to target the *gdhA* gene. Using two properly designed sgRNAs that repress the same gene, the overall silencing efficacy can be improved to > 1000 fold (**c**), but when two sgRNAs overlap, either on the same stand or not, the knockdown effect is suppressed (**a** and **b**). Experiments were performed on at least three biological repeats

Properly designed multiple sgRNAs can improve repression efficiency in *E. coli* (Qi et al. 2013). To test whether CRISPRi silences gene transcription more efficiently with multiple sgRNAs, we designed pairs of sgRNAs: g12 and g13, g12 and g14, and g12 and g15, targeting different loci on the *gdhA* gene (Fig. 5). Next, we cloned g12 into pJT4, under the control of the Trc promoter, and cloned g13, g14, and g15 downstream of the pJ23119 promoter. Finally, we introduced these plasmids into *V. parahaemolyticus* and examined the repression efficacies. G12 and g15 targeted well-separated sequences. Combining these two sgRNAs improved silencing efficacy ~ 1200-fold (Fig. 5 c). The sequences g12 and g13 overlapped on the same strand, while the sequences g12 and g14 overlapped on different strands. The combinatorial silencing effects of using these two double-sgRNA pairs are suppressive (Fig. 5a, b). Taken together, our results show that appropriately positioned two sgRNAs (g12 and g15) targeting the same gene improved silencing efficacies, and the combination of overlapping (or colliding) sgRNAs resulted in suppressive effects.

We also examined the gene transcript level of *pyrD*, the gene immediately downstream of *gdhA*, to explore the effect exerted by silencing the upstream gene *gdhA* with sgRNA g6. The results showed that although the *gdhA* transcript level was significantly reduced (SI Fig. 4), the *pyrD* transcript level only slightly decreased. These results suggested that silencing the upstream gene *gdhA* did not reduce the *pyrD* transcript level and that the promoter of *pyrD* drove most of the *pyrD* transcription, which indicated that the CRISPRi silencing effect was exclusively on the gene targeted.

### Depletion of an essential gene in *Vibrio parahaemolyticus* by CRISPRi

**Fig. 6:**
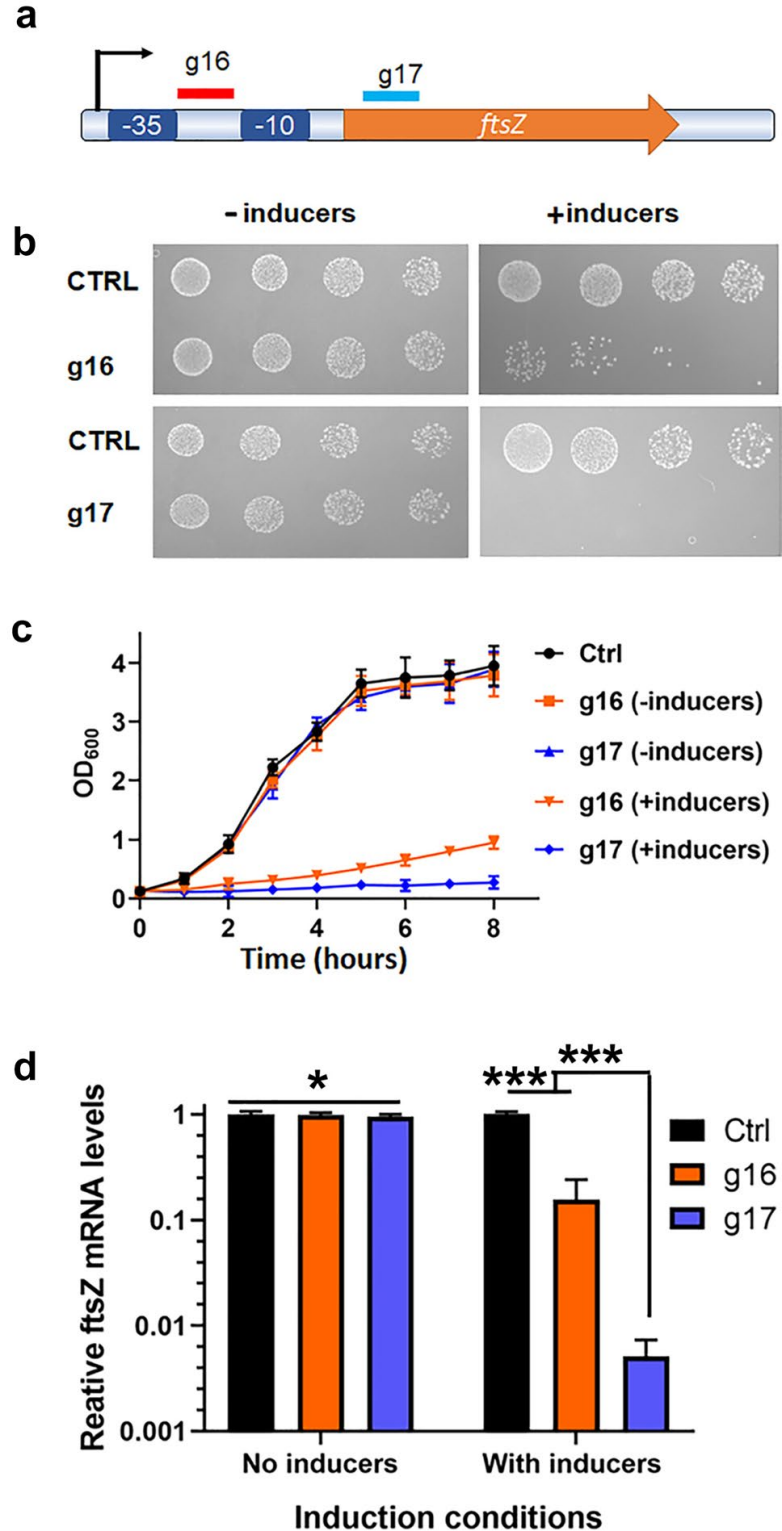
CRISPRi suppresses the essential gene *ftsZ* effectively. **a** sgRNA g16 targets the sequence between the − 35 box and − 10 box of *ftsZ* on the non-template strand. sgRNA g17 targets the non-template strand of the *ftsZ* gene. **b** Bacterial spotting test sgRNA g16 and g17 repression. The bacteria were diluted fivefold in each spot. sgRNA g16 did not completely inhibit bacterial growth, but sgRNA g17 inhibited bacterial growth. **c** Growth curve showing g16 and g17 inhibited bacterial growth. **d** The transcription level of *ftsZ* was determined by RT-qPCR. IPTG and arabinose induced dCas9 and sgRNA expression and repressed *ftsZ* gene transcription. *P > 0.05, ***P < 0.05. Experiments were performed with at least three biological repeats. A *t* test was used to examine the significance

Although gene knockout is a classic technique utilized in genomic functional studies, the knockout of an essential gene is lethal to organisms. Therefore, conditional gene silencing is an alternative. CRISPR interference has been demonstrated to be a powerful tool in genomic function studies in bacteria (Qi et al. 2013). The *ftsZ* gene encodes a ring-forming protein essential for cell division. This ring regulates the timing and location of cell division. Depleting the FtsZ protein disrupts cell duplication and impairs bacterial growth (Tan et al. 2018). Herein, we designed sgRNAs g16 and g17, targeting the promoter region and coding region of *ftsZ*, respectively. Then, we cloned them into the pJT3 vector and transformed them into *V. parahaemolyticus* (Fig. 6a).

*V. parahaemolyticus* containing the pJT3-g16 plasmid and pJT3-g17 plasmid were inoculated and shaken overnight; bacterial cultures were diluted progressively and spotted on LB agar plates with or without inducers, i.e., 2 mg/ml arabinose and 0.5 mM IPTG, and incubated at 37°C overnight. No *V. parahaemolyticus/pJT3-g17* colony grew on plates with inducers, suggesting that *ftsZ* transcription was tightly repressed. However, *V. parahaemolyticus/pJT3-g16* colonies grew on plates with inducers, and the magnitudes were two orders less than those grown on plates without inducers (Fig. 6b), indicating leaky FtsZ expression.

The overnight cultures were also expanded 1:100 in 25 ml LB, with or without inducers, i.e., 2 mg/ml arabinose and 0.5 mM IPTG, and shaken at 37°C at 250 rpm. Bacterial growth was monitored by measuring the OD_600_, and the transcript levels of *ftsZ* were evaluated. The results showed that the *V. parahaemolyticus*/pJT3-g16 growth rate decreased by ~ 8.5-fold, while no noticeable *V. parahaemolyticus*/pJT3-g17 growth was observed after adding inducers (Fig. 6c). More than 99.5% transcription of *ftsZ* was repressed in *V. parahaemolyticus*/pJT3-g17 grown in LB with inducers (Fig. 6d) upon adding inducers. However, there was still 15% *ftsZ* mRNA after adding inducers to *V. parahaemolyticus*/pJT3-g16 culture, showing that this small leakiness enabled bacteria to grow slowly on plates and in liquid media (Fig. 6b, c).

**Fig. 7:**
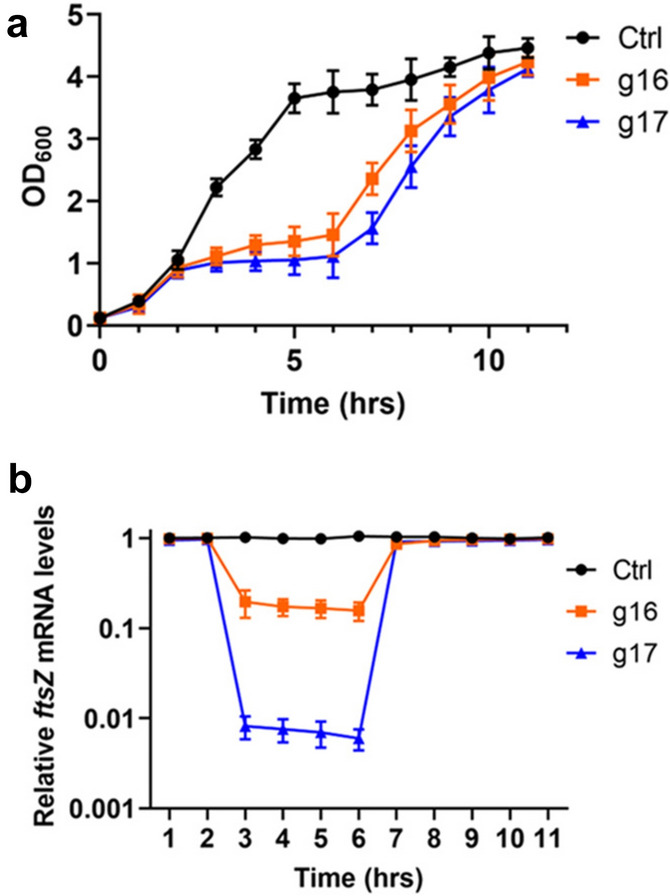
CRISPRi repression of *ftsZ* is reversible. **a** Growth curves of bacteria with ftsZ repressed. Bacterial growth (g16 and g17) was inhibited after inducers were added, and the growth recovered when inducers were removed. **b** RT-qPCR was used to examine *ftsZ* mRNA level along bacterial growth. The mRNA levels of *ftsZ* decreased after inducers were added and recovered after inducers were removed. Experiments were performed with at least three biological repeats

We also monitored the dynamic growth rates and transcription levels. First, the three bacteria, *V. parahaemolyticus*/pJT3-g16, *V. parahaemolyticus*/pJT3-g17, and the control strain (V. parahaemolyticus/pJT3), were inoculated and grown overnight. These cultures were expanded 1:100 in 25 ml LB medium without inducer for the first 2 h. They grew at the same rate (Fig. 7a), indicating that the *ftsZ* gene in all three bacteria was not repressed, which was confirmed in RT-qPCR assays (Fig. 7b). In the next four hours, inducers (0.5 mM IPTG and 2 mg/ml arabinose) were added to the cultures. Compared to the control culture (at the corresponding time point), *V. parahaemolyticus/*pJT3-g16 slowed the growth rate, and *V. parahaemolyticus/*pJT3-g17 almost stopped growing. The *ftsZ* transcript levels were in line with the different growth rates; the mRNA level of the control strain remained unchanged. However, the mRNA level of *V. parahaemolyticus/*pJT3-g16 decreased by ~ 83%, and the mRNA level of *V. parahaemolyticus*/pJT3-g17 was reduced to 0.7% of that of the control samples (Fig. 7 b). Finally, the bacteria were pelleted and resuspended in LB medium without induction and continued to grow for 5 h. The growth rates of *V. parahaemolyticus/*pJT3-g16 and *V. parahaemolyticus/*pJT3-g17 recovered, and the end-point OD_600_s were similar to that of the control, indicating that the CRISPRi-mediated repression was removed, and *ftsZ* mRNA transcription resumed, which was confirmed by RT-qPCR (Fig. 7). Taken together, the repression mediated by the pJT3 CRISPRi system is titratable, precise, and reversible.

## Discussion

In this research, we successfully developed a new CRISPRi system based on the combination of CRISPR/dCas9 and the shuttle vector pVv3 to repress gene expression in *V. parahaemolyticus*. We inserted a P_Bad_ promoter driving the CRISPR/dCas9 expression cassette into a single plasmid. The P_BAD_ promoter is precise, sensitive, tightly controlled, and active upon the addition of arabinose (Guzman et al. 1995). The Trc promoter is a hybrid of the Lac promoter (Reznikoff 1992) and the Trp promoter (Cohen & Jacob 1959), whose transcription can be induced by IPTG. Therefore, gene silencing can only be implemented upon the addition of both arabinose and IPTG, guaranteeing tight control of induced gene repression. The silencing complex sgRNA:dCas9 ribonucleoprotein can be induced by modest concentrations of inducers, i.e., 0.5 mM IPTG and 2 mg/ml arabinose, which facilitates its applications. However, mRNA levels are not always proportional to protein levels in the assays. For example, when *lacZ* and *gdhA* mRNA levels were silenced and reduced to less than 5% of the control, the enzymatic activities of β-galactosidase and glutamate dehydrogenase were decreased to 10% of the control. This inconsistency may be due to several factors, such as mRNA translation efficiency, stability, and reaction mixture variation.

Using the correct sgRNA is crucial for effective CRISPRi-mediated silencing. Therefore, we optimized the sgRNA in this research. sgRNAs targeting the promoter region and those targeting the protein-coding sequence had different effects in this study. Transcription starts with the association of RNA polymerase (RNAP) and transcription factors (TFs) with the promoter region, leading to the formation of a transcription complex and initiating transcription. Therefore, the Cas9:sgRNA complex binding to the proper promoter region would sterically prevent RNAP and transcription factors from associating with the DNA motif, thus blocking transcription initiation and efficient gene repression. sgRNA g2 directed the dCas9:sgRNA to the center of the promoter sequence and knocked down *gdhA* transcription significantly, while sgRNAs g3 or g4, both of which targeted in the proximity of promoter, suppressed *gdhA* gene expression slightly. This result confirmed that the binding of dCas9:sgRNA g2 to the center of the promoter sterically obstructed the association of RNAP and transcription factors much more intensely than those of dCas9:sgRNA g3 and g4, which bound to the periphery of the promoter. As expected, binding of dCas9:sgRNA g5 163 bp upstream of TSS did not impede the attachment of RNAP and TF binding to the promoter, resulting in inefficient gene regulation.

In the transcription elongation stage, RNAP unwinds double-strand DNA and produces nascent mRNA that pairs with the template strand. The newly synthesized mRNA, RNAP, and DNA form a transcription bubble. The bubble proceeds forward until transcription finishes or halts. Therefore, silencing *gdhA* with sgRNAs targeting different DNA strands may yield distinct effects. Silencing *gdhA* with sgRNA g6, g8, and g10 only slightly suppressed gene expression, indicating that RNAP could read through despite the binding of dCas9:sgRNA complexes to the template strand. This may have resulted from the fact that the pairings of template strand DNA–sgRNA were unzipped by the helicase of RNAP, given that the sgRNAs faced the RNAP, and then the RNAP moved through the targeting sites, as Qi et al. proposed previously (Qi et al. 2013). In contrast, when silencing *gdhA* with sgRNAs g7, g9, and g11, which directed dCas9:sgRNA complexes to the non-template strand, *gdhA* gene expression was significantly suppressed. One possible mechanism is that RNAP did not unwind the non-template DNA and sgRNA pairings since the sgRNAs did not face the RNAP. Thus, the transcription complex moved forward and collided with the dCas9:sgRNA complex at the sgRNA targeting site; consequently, transcription paused, and *gdhA* expression was repressed. Another possible mechanism is that the dCas9:sgRNA binds to the nascent mRNA, leading to mRNA degradation as RNAi does. However, an earlier report demonstrated that the pausing site is a 19-base pair by sequencing the 3′ ends of nascent transcripts associated with RNA polymerase when CRISPRi in *E. coli*, which is perfectly consistent with the 18 bp distance between the nucleotide incorporation site of RNAP and its front edge, thereby strongly supporting the point-of-view that physical collision between RNAP and the dCas9:sgRNA complex bound on the non-template strand brought about gene repression. (Qi et al. 2013). The suppression efficacies were inversely correlated with the distances between the translation initiation site and the sgRNA target sites. This may be due to the varying RNAP kinetics in different elongation phases.

In conclusion, properly designed sgRNAs targeting the promoter center and the non-template strand DNA of the protein-coding region effectively repress gene expression, and sgRNA targeting sequences closer to the translation starting site have the highest efficiency in gene knockdown. Our results align with previous findings (Qi et al. 2013), suggesting a possible universal rule for CRISPRi sgRNA design.

Gene repression by this CRISPRi system is tunable and invertible. Our results demonstrated that effective gene repression occurs only when appropriate amounts of arabinose and IPTG are available in the culture medium, and the gene repression can be reversed by removing the inducers. Furthermore, the repression effect could be precisely tuned by adjusting the amounts of those two inducers, suggesting its great potential for use in functional genomic studies. In addition, we cloned another pJ23119 driving the sgRNA expression cassette into this CRISPRi system to express dual sgRNAs in a single plasmid pJT4. With this plasmid, we demonstrated improved gene silencing with two properly designed and independently expressed sgRNAs, showing the potential to express two sgRNAs and knock down two genes simultaneously. This can expand its application in genomic functional studies of *V. parahaemolyticus*.

Gene knockout is routinely used in genomic functional studies but is not feasible for genomic functional studies of essential genes in organisms. In this research, we demonstrated the power of this CRISPRi system in the study of essential genes using *ftsZ* as an example. FtsZ is vital for bacterial growth; depletion of this protein stops *V. parahaemolyticus* growth. In the experiment, adding inducers repressed *ftsZ* transcription and slowed/stopped bacterial growth on plates and in liquid media. Although sgRNA g17 tightly repressed *ftsZ* expression, sgRNA g16 only knocked down approximately 85% of ftsZ expression. This leaky expression shows that appropriately designed sgRNA is the pivotal factor for efficient CRISPRi. The GC content of sgRNA g16 was 40%, merely reaching the lower limit of “optimal GC content 40–60% (Ren et al. 2014), which may be one reason for the leakiness. Another possible cause is that this sgRNA transcript has a motif ‘GUUG’ that may form a hairpin structure or self-anneal with another motif ‘CAAC’ in the gRNA scaffold sequence, thereby preventing sgRNA from pairing with the targeting site efficiently. These FtsZ depletions could be reversed by removing inducers. The bacteria then resumed growth, showing that this system can silence gene expression in a rapid, precise, tuneable, and reversible way, thereby enabling the temporary and dynamic depletion of the essential protein of interest and revealing great potential in the functional study of crucial genes in *V. parahaemolyticus*.

In this research, we developed a highly efficient CRISPRi system that repressed gene expression in a reversible, timing-controllable, and protein amount-tuneable way in *V. parahaemolyticus*. This system will promote studies of bacterial physiology, pathogenesis, virulence factor identification, and the interaction between this pathogen and hosts. Based on this system, scientists can design and prepare a CRISPRi library that targets and knockdown every gene in *V. parahaemolyticus*, thereby interrogating each gene, performing CRISPRi-seq for genome-wide fitness quantification in this bacterium, identifying essential genes, and discovering new drug targets.

## Materials and methods

### Bacterial strains and cultivation

*E. coli* DH10B was used for plasmid cloning and maintenance. *E. coli* strains were cultured in LB medium at 37 °C with 100 μg/ml ampicillin and 50 μg/ml kanamycin antibiotics where necessary. The *Vibrio parahaemolyticus* RIMD 2210633 strain was used in this study. For CRISPRi repression experiments, cultures were induced with 0.5 mM IPTG and 2 mg/ml arabinose unless otherwise noted.

### Construction of pVv3-derived pCRISPRi vectors

The DNA fragment (1798–3107) containing Origin of Replication of pVv3 (NCBI accession # HG326273) was chemically synthesized (Tsingke biology, Nanjing, China) with BbvCI site at 5’-end and NcoI site at 3’-end, and cloned in pBAD18-Kan (ATCC Cat#87,397) between BbvCI and PciI to replace pBR322 Origin, generating plasmid pJT1, then inactivated Cas9 protein-coding gene was PCR-amplified from pdCas9-bacteria (Addgene #44,249) with primer dCAS9for: atatattctagaAAAGAGGAGAAAGGATCTATGGATAAG and dCAS9rev: atatatgtcgacTTAGTCACCTCCTAGCTGACTC using Q5 DNA polymerase (NEB Cat#0491S) and cloned in pJT1 between NheI and SalI, forming plasmid pJT2, sgRNA transcription cassette containing Trc promoter (TTGACAATTAATCATCCGGCTCGTATAATGTGTGgcaggtgGCGAGACCATTGGTcacctgcCTCAGTTTTAGAGCTAGAAATAGCAAGTTAAAATAAGGCTAGTCCGTTATCAACTTGAAAAAGTGGCACCGAGTCGGTGCTTTTTT) was synthesized (Tsingke biology, Nanjing, China, TRC promoter sequence is underlined and sgRNA scaffold is *italic*), and cloned in pJT2 between EcoO109I and SgrAI, the DNA fragment-coding sgRNAs were designed with CHOPCHOP (https://chopchop.cbu.uib.no/) and cloned between PaqCI sites, producing pJT3 plasmids (Plasmid sequences file in the supplementary materials). To prepare the dual sgRNA-expressing vector, a pJ23119-driving sgRNA transcription cassette was cloned into the NarI site.

### Plasmid methylation with *Vibrio parahaemolyticus* cell extract

To overcome restriction modification and improve the transformation efficiency of CRISPRi (pJT3) plasmids into *V. parahaemolyticus*, we treated pJT3 plasmids with *V. parahaemolyticus* cell extract to methylate plasmid DNA. Plasmids were isolated from *E. coli* using the Qiagen Plasmid Midi Kit (Cat#12,145) following the manufacturer’s instructions. First, 100 μg pJT3 plasmids were incubated with 100 μL (containing ca. 350 μg V*. parahaemolyticus* cell extract protein) in methylation reaction buffer (20 mM Tris–acetate (pH 7.9), 50 mM potassium acetate, 5 mM Na_2_EDTA, 1 mM DTT and 200 mM S-adenosyl-L-methionine) at 37 °C for 30 min. Then, following the manufacturer’s instructions, the plasmid DNA was purified with a Qiagen mini-preparation kit (Cat#12,125).

After methylation and DNA purification, the plasmid was subjected to restriction enzyme SalI (NEB, cat#R3138S) digestion, and reaction mixtures were analyzed by 1% agarose gel electrophoresis. The gel was then stained with ethidium bromide (Merck, Cat#E8751) and imaged on a UV Gel Doc XR + System (Bio-Rad).

### Reverse transcription-quantitative real-time PCR

Total RNA was extracted using hot phenol–chloroform. Total RNA samples were then treated with DNase I (NEB, Cat#M0303S), and equal amounts of RNA were reverse transcribed to cDNA using the Transcriptor First Strand cDNA Synthesis Kit (Roche, Cat#04379012001) according to the manufacturer’s instructions. Real-time quantitative PCR of the *lacZ*, *gdhA,* and *pyrD* genes was then performed using the following primer sets: lacZfor (GGTTGCCATGACCGCTTTAT) and lacZrev (CGCTCATCCAATTCTGGCAA) gdhAfor (TGAACTGCTACAAGCGAACG) and gdhArev (CCATGCAGCTGAAGAAACGA), pyrDfor (GATGCGATGGGATTTGGCTT) and pyrDrev (GTTGATGATGCCTTCGGCTT) and LightCycler FastStart DNA Master SYBR Green I (Roche, Cat#12,239,264,001) on an ABI 7500 real-time PCR system (Applied Biosystems, Beverly, MA). Reported transcript levels are the averages of biological triplicates measured in technical duplicates.

### *V. parahaemolyticus* transformations

*V. parahaemolyticus* transformations were performed by electroporation according to the method described by Klevanskaa K. et al. with minor modifications (Klevanskaa et al. 2014). Briefly, a single bacterial colony was inoculated in LB broth and shaken overnight at 37 °C. The overnight cultures were diluted 1:100 in 20 ml LB broth and shaken at 37 °C in a 250 ml flask until they reached an OD_600_ of ca. 0.6. The cells were pelleted (2500 g; 15 min; 4 °C) and resuspended in 25 ml of ice-cold resuspension Buffer (1 mM Mes-NaOH buffer, pH 6, supplemented with 200 mM sucrose). The cells were washed twice and resuspended in 200 μL of water. One hundred microliters of the cell was mixed with ca.300 ng of methylated plasmid DNA. Electroporation was performed using a Bio-Rad MicroPulser (Bio-Rad, USA) with 1 mm gap electroporation cuvettes (4–5.6 ms pulse duration; 1.8 kV pulse). Transformed cells were recovered in 1 ml of SOC medium for 1.5 h at 37 °C, and the bacteria were plated on LB agar with appropriate antibiotics and incubated at 37 °C for 16 h.

### β-D-galactosidase activity assay

β-D-galactosidase activity assays were performed as Tan SZ et al. described with minor modifications (Tan et al. 2018). Briefly, overnight, *V. parahaemolyticus* cultures were expanded 1:100 in 100 ml LB with or without appropriate amounts of inducers (IPTG and arabinose) and shaken at 37℃, 250 rpm. The cells were harvested at an OD_600_ of ca. 0.8 and lysed in 5 ml PBS buffer with protease inhibitor cocktail (Roche Cat#11,697,498,001) using a Sonicator (Fisher) on ice-water mix. The lysates were centrifuged at 20,000 g for 30 min at 4℃ and supernatants were collected for activity assays. According to the manufacturer’s instructions, the total protein concentration was determined using a BCA protein assay kit (Beyotime, Beijing, Cat#P0011). Whole β-galactosidase activity assays of the lysates were determined by measuring the initial rate of the enzyme-catalyzed break of orthonitrophenyl-β-galactoside (ONPG). The absorbance change of OD_420_ of reaction mixtures was measured, and the enzymatic activity was estimated as the rate of change of A_420_ normalized by the total protein amount in the assays.

### NADP-dependent glutamate dehydrogenase assay

According to the manufacturer's instructions, NADP-dependent glutamate dehydrogenase activity was measured using a Glutamate Dehydrogenase (GDH) Activity Assay Kit (Abcam, ab102527). Briefly, the cells were lysed in PBS buffer, and the lysates were high-speed centrifuged; supernatants were collected, and the total protein concentrations were determined using a BCA Kit as described in an earlier section; the enzymatic activities that GDH consumes glutamate and generates NADH were quantified colorimetrically by measuring the absorbance change of OD_450_; and the relative enzymatic activities were normalized to the protein amount in the reaction mixtures.

### Growing bacteria with essential gene depletion on plates

Cells transformed with pJT3 plasmids were recovered in LB medium and plated in LB agar plates for 16 h. Next, the single colonies were inoculated in 5 ml LB and shaken overnight at 37 °C. The overnight cultures were appropriately diluted and spotted on LB agar with and without 0.5 mM IPTG and 2 mg/ml arabinose. Finally, plates were incubated at 37 °C for 16 h before imaging and quantification.

### Supplementary Information

Below is the link to the electronic supplementary material.

Supplementary file1 (TIF 3627 KB)

Supplementary file2 (TIF 4034 KB)

Supplementary file3 (TIF 467 KB)

Supplementary file4 (TIF 1845 KB)

Supplementary file5 (DOCX 28 KB)

## Data Availability

Not applicable.
