# Supplementary figures and images for: A robust CRISPR interference gene repression system in Vibrio parahaemolyticus
Source: Arch Microbiol. 2023 Dec 26;206(1):41. doi: 10.1007/s00203-023-03770-y (PMC10751265; doi:10.1007/s00203-023-03770-y)

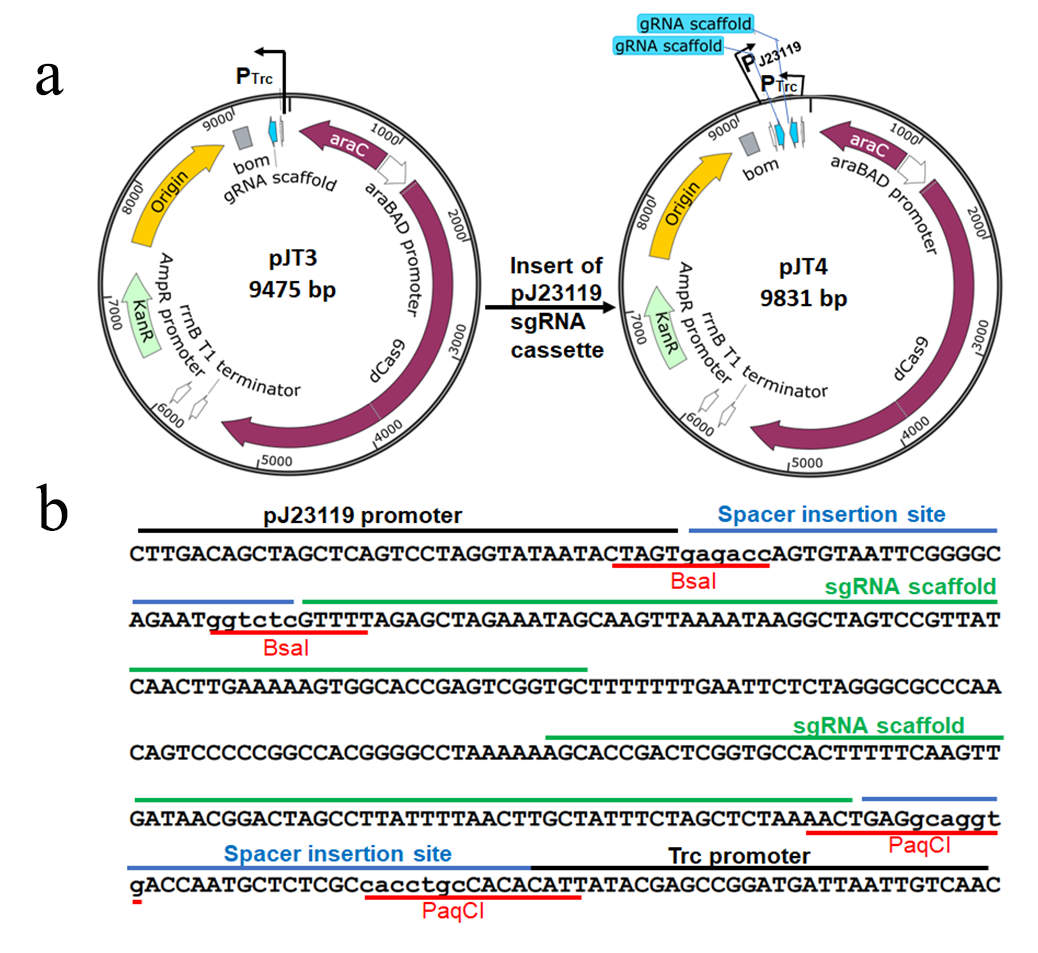

Supplement: Supplementary file 1 — Supplementary file1 (TIF 3627 KB) [file 203_2023_3770_MOESM1_ESM.tif]

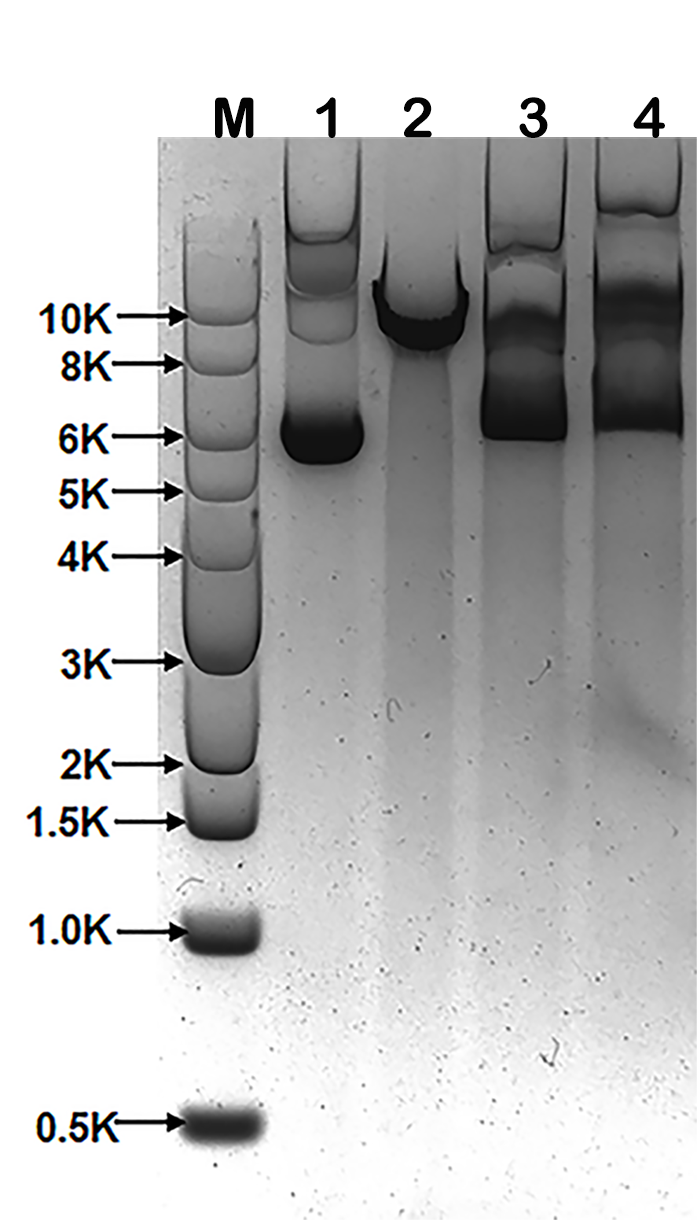

Supplement: Supplementary file 2 — Supplementary file2 (TIF 4034 KB) [file 203_2023_3770_MOESM2_ESM.tif]

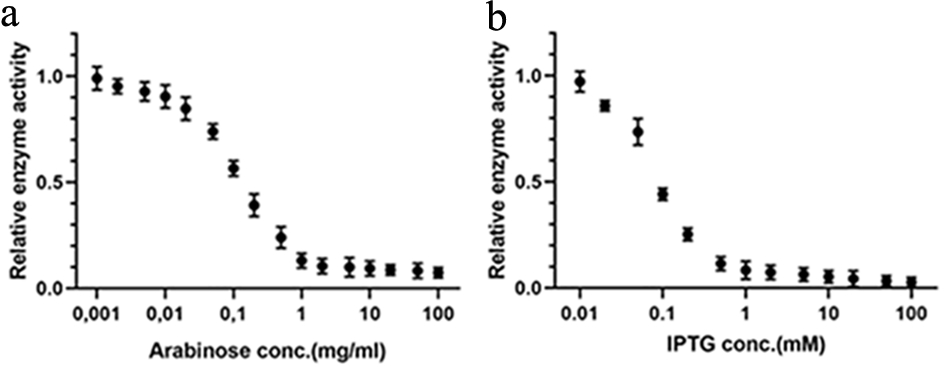

Supplement: Supplementary file 3 — Supplementary file3 (TIF 467 KB) [file 203_2023_3770_MOESM3_ESM.tif]

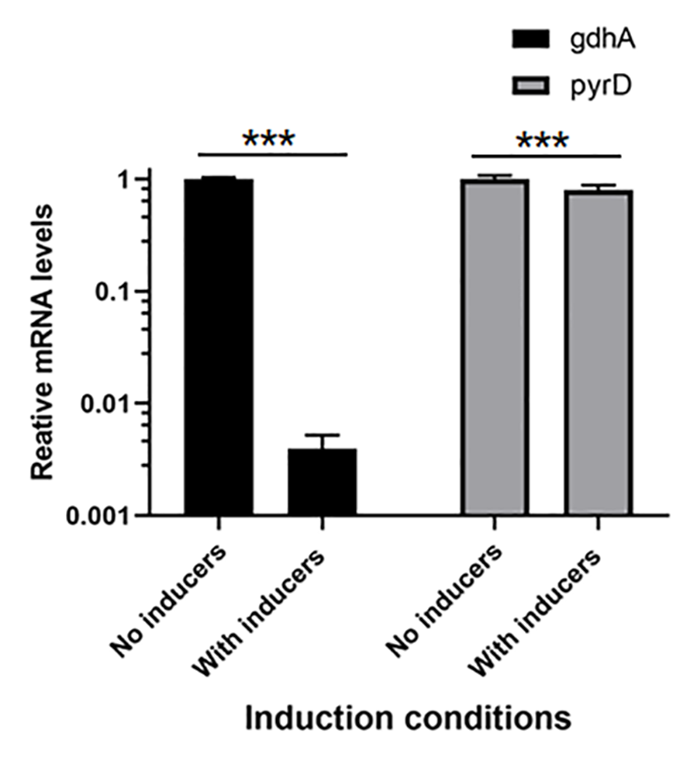

Supplement: Supplementary file 4 — Supplementary file4 (TIF 1845 KB) [file 203_2023_3770_MOESM4_ESM.tif]
