# Supplementary material for: A robust CRISPR interference gene repression system in Vibrio parahaemolyticus
Source: Arch Microbiol. 2023 Dec 26;206(1):41. doi: 10.1007/s00203-023-03770-y (PMC10751265; doi:10.1007/s00203-023-03770-y)
Supplement: Supplementary file 5 — Supplementary file5 (DOCX 28 KB) [file 203_2023_3770_MOESM5_ESM.docx]

**SI Table 1**

| **sgRNAs** | **Targeting sequences on the bacterial chromosome** | **purposes** | **Targeting**  **location** |
| --- | --- | --- | --- |
| g1 | CATAAGCGCACCAAGCCGAG | repress β-galactose expression | promoter |
| g2 | ACAAAACAACAATTCTAATG | repress *gdh* expression | promoter |
| g3 | TTACCTATGCTTAGTTTCTA | repress *gdh* expression | non-template strand |
| g4 | TTACCCTAGAAACTAAGCAT | repress *gdh* expression | non-template strand |
| g5 | GTATTTGCAGAGAGTTAAAT | repress *gdh* expression | template strand |
| g6 | CGCGCGTGAAAATGTAGTGC | repress *gdh* expression | non-template strand |
| g7 | CGGCACTACATTTTCACGCG | repress *gdh* expression | template strand |
| g8 | TTGCACGTGGTGGCTTACGT | repress *gdh* expression | non-template strand |
| g9 | TTTCTGTACGGAAGTCTTCT | repress *gdh* expression | template strand |
| g10 | CTCGGTAGCTCTTCGTGAAC | repress *gdh* expression | non-template strand |
| g11 | ACGAAGAGCTACCGAGAATT | repress *gdh* expression | template strand |
| g12 | ACGTCTATCGAGCCTTCAAT | repress *gdh* expression | non-template strand |
| g13 | ATCGAGCCTTCAATCGGCAC | repress *gdh* expression | non-template strand |
| g14 | AAATATCCAGTGCCGATTGA | repress *gdh* expression | template strand |
| g15 | GAATTGCTCTTCAAACGAGT | repress *gdh* expression | non-template strand |
| g16 | AACCTGAGTTGCAGGATAAA | repress *ftsZ* expression | promoter |
| g17 | TGGTAACGCCGTTGAACACA | repress *ftsZ* expression | non-template strand |
